# Effects of unilateral robotic limb loading on gait characteristics in subjects with chronic stroke

**DOI:** 10.1186/1743-0003-7-23

**Published:** 2010-05-21

**Authors:** Ira Khanna, Anindo Roy, Mary M Rodgers, Hermano I Krebs, Richard M Macko, Larry W Forrester

**Affiliations:** 1 Department Physical Therapy and Rehabilitation Science, University of Maryland School of Medicine, Baltimore, Maryland, 21201, USA; 2 Department of Mechanical Engineering, Massachusetts Institute of Technology, Cambridge, Massachusetts, 02139, USA; 3 Research Rehabilitation and Development, Baltimore Veterans Affairs Medical Center, Baltimore, Maryland, 21201, USA; 4 Department of Neurology, University of Maryland School of Medicine, Baltimore, Maryland, 21201, USA; 5 Department of Neurology and Neuroscience, Weill Medical College of Cornell University, New York, New York, 10021, USA; 6 Department of Medicine, University of Maryland School of Medicine, Baltimore, Maryland 21201, USA

## Abstract

**Background:**

Hemiparesis after stroke often leads to impaired ankle motor control that impacts gait function. In recent studies, robotic devices have been developed to address this impairment. While capable of imparting forces to assist during training and gait, these devices add mass to the paretic leg which might encumber patients' gait pattern. The purpose of this study was to assess the effects of the added mass of one of these robots, the MIT's Anklebot, while unpowered, on gait of chronic stroke survivors during overground and treadmill walking.

**Methods:**

Nine chronic stroke survivors walked overground and on a treadmill with and without the anklebot mounted on the paretic leg. Gait parameters, interlimb symmetry, and joint kinematics were collected for the four conditions. Repeated-measures analysis of variance (ANOVA) tests were conducted to examine for possible differences across four conditions for the paretic and nonparetic leg.

**Results:**

The added inertia and friction of the unpowered anklebot had no statistically significant effect on spatio-temporal parameters of gait, including paretic and nonparetic step time and stance percentage, in both overground and treadmill conditions. Noteworthy, interlimb symmetry as characterized by relative stance duration was greater on the treadmill than overground regardless of loading conditions. The presence of the unpowered robot loading reduced the nonparetic knee peak flexion on the treadmill and paretic peak dorsiflexion overground (p < 0.05).

**Conclusions:**

Our results suggest that for these subjects the added inertia and friction of this backdriveable robot did not significantly alter their gait pattern.

## Background

Over 795,000 strokes occur in the United States each year [1]. Of those individuals that survive, approximately two-thirds have residual motor deficits, including impaired gait [1]. Lower extremity hemiparesis has been shown to reduce walking speed and endurance [2-6] as well as gait parameters such as step length [4,7] and stance duration [8]. Studies have shown that impaired swing initiation, abbreviated paretic single limb support [9,10], decreased hip flexion, increased knee flexion, and increased ankle plantarflexion at toe off [8] are all characteristic of hemiparetic gait.

Rehabilitation intervention has demonstrated significant potential for improving motor function and gait [3,11]. Traditional models of gait rehabilitation often employ task-oriented exercises such as stepping and weight shifting [12] as well as manual stretching to increase range of motion and strength training [13]. Recent studies have shown that practice over a treadmill can improve cardiovascular fitness and ambulatory performance in individuals with hemiparetic gait [13-16] including improved interlimb symmetry [15], cadence and gait velocity [3,14,17,18]. A recent Cochrane Report has reported that along with treadmill training, there is evidence to suggest electromechanical gait training may improve independent walking [19].

The Cochrane Report includes results observed in trials with two devices, namely the Gait Trainer I and the Lokomat^®^. More recent studies have focused on robotic devices for the ankle joint [20] to address the problem of drop foot that occurs during hemiparetic gait [21]. For this class of robotic devices, a possible confounding factor resulting from wearing the device during walking is the added mass which might encumber patients' ability to move, especially their leg during walking. Noble and Prentice found that adding a 2 kg weight to the non-dominant leg of young adults resulted in increased knee and hip flexion during the swing phase as well as reduced plantarflexion at toe off of the weighted limb [22]. Since individuals with hemiparesis have asymmetric gait, the addition of asymmetric loading to the paretic limb may further increase asymmetry and affect gait kinematics. For example, one study of individuals with post-stroke hemiparesis showed that adding a unilateral weight to the nonparetic leg increased hip and knee excursions in the paretic limb [23].

Here we examined the effects of asymmetric or unilateral loading of the paretic limb during task-oriented gait therapy. Specifically, we sought to assess the effects of the added inertia and friction of unpowered ankle robot on gait parameters, interlimb symmetry, and lower extremity joint kinematics in chronic stroke survivors. We examined these effects in two common rehabilitation training scenarios: walking over ground (OG) and treadmill (TM) training. We hypothesized that loading the paretic limb with the robot would change bilateral joint kinematics.

## Methods

### Subjects

Ten chronic stage stroke survivors (4 males and 6 females) were recruited through the Veterans Affairs (VA) Maryland Exercise and Robotics Center of Excellence and the University of Maryland (UM) Claude D. Pepper Older Americans Independence Center in Baltimore, Maryland. Participants met the following inclusion criteria: a) at least 6 months post ischemic stroke with residual hemiparetic gait, b) able to walk on a treadmill, and c) had a score greater than a 23 on the mini mental state exam (MMSE) [24] and able to follow two step commands. Individuals with unstable angina, congestive heart failure within the last 3 months, major orthopedic or chronic pain, poorly controlled hypertension, recent hospitalization for severe disease or surgery, a history of severe ankle injury or severe receptive aphasia were excluded from the study. The study was approved by the VA Rehabilitation Research and Development (RR&D) Committee and UM Institutional Review Board, and Massachusetts Institute of Technology's Committee on the Use of Humans as Experimental Subjects (COUHES). All participants signed informed consent and underwent medical evaluations to establish eligibility.

### Apparatus

**Figure 1:**
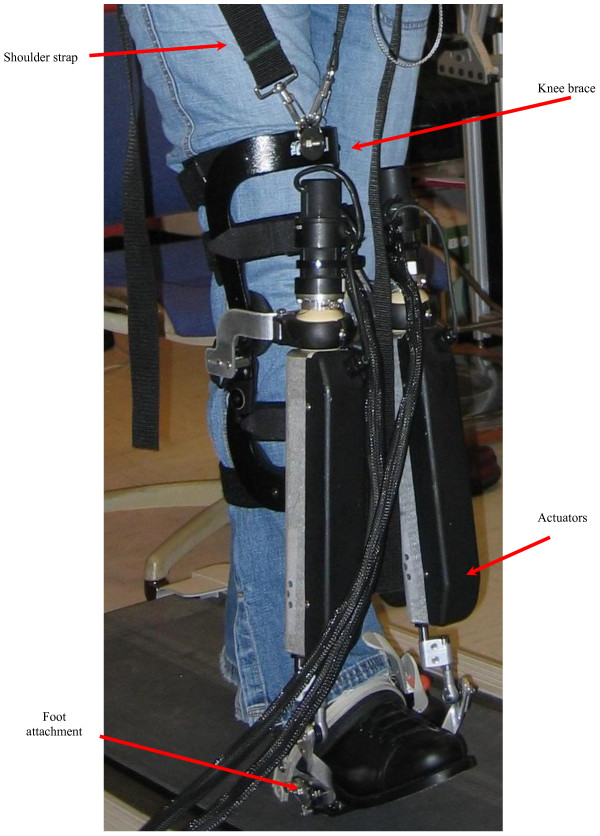
**Anklebot**. The figure identifies the main components of the device.

The robot used in this study is a backdriveable or low end-point impedance device that allows mobility at the ankle joint in all three degrees of freedom (DOFs) but actuates the ankle in only two of those three DOFs, namely dorsi/plantarflexion and inversion/eversion (Figure 1). The anklebot weighs 3.6 Kg and has low static friction (<1 N-m). It is mounted proximally to the leg and anterior to the shank to minimize perception of loading [25]. It attaches to the subject's paretic limb by way of an orthopedic knee brace (Townsend Design, Bakersfield, CA) that is affixed to the thigh and shank by multiple anterior and posterior Velcro straps, each positioned to match the natural contour of the distal thigh and proximal shank segments. Pads protect the medial and lateral condyles, where hinges approximate joint axes of rotation. Orthopedic shoes with steel shank construction secure the robot's distal attachment via quick-release mechanisms as well as a single strap over the proximal metatarsals. The robot connects to the knee brace proximally via a set of quick-release locking clamps. An adjustable shoulder strap that connects to the knee brace provides additional anti-gravity support during the swing phase of walking. Design and performance characteristics of the anklebot have been described elsewhere [20].

### Procedure

Clinical assessments included a medical history, MMSE, and active and passive ranges of motion (AROM and PROM) [26] of the paretic hip, knee, and ankle joints. In addition, spasticity for the paretic knee and ankle was assessed using the Modified Ashworth Spasticity (MAS) Scale [27]. Manual Muscle Testing (MMT) [28] was performed for the hip, knee and ankle flexor and extensor muscle groups. Body mass index (BMI) was also calculated for each participant.

Two separate sessions were scheduled to evaluate baseline walking performance over ground and on the treadmill. The first session assessed participants' ability to walk independently overground (OG) and on the treadmill (TM). The session began with three 10-meter walks to determine participants' self-selected floor walking velocity using an instrumented gait mat (GAITRite^®^, CIR Systems, Havertown, Pa). We determined and attempted to control the speed for all the following conditions from this self-selected floor walking velocity. Participants, then, walked OG and then on a TM (6 trials per condition with TM trials lasting 15 seconds each). To minimize fatigue, participants were asked to return for a second session two days later when they repeated OG and then TM walking while wearing the unpowered anklebot on the paretic leg ("OGR" and "TMR" conditions, respectively). When walking on the treadmill, individuals were instructed to hold the handrails. Optimal fitting of the anklebot was established for each participant prior to walking to minimize its slippage during gait and to maximize subject comfort. Optotrak^® ^motion analysis system (Northern Digital Inc., Waterloo, Canada) was utilized along with the MotionMonitor™ computer software to collect gait kinematics. Light emitting diodes (LED) were attached to the sacrum and the posterior mid-thigh, the posterior mid-gastrocnemius, and lateral aspect of the foot of each leg for 3-D motion analysis. Footswitches (Myopac Jr., Run Technologies, Mission Viego, CA) were placed inside the subjects' shoes to determine timing of initial contact and the end of pre-swing in the gait cycle. Before overground and treadmill trials, individuals were asked to stand with feet shoulder width apart to collect neutral stance measurement. The Lumbosacral (L5/S1) joint, bilateral anterior superior iliac spine (ASIS), knee joints, ankle joints, and feet positions were captured during the neutral stance measurement. All kinematic variables were expressed with respect to this neutral stance or "zero" angle. Participants were instructed to walk across a 7.3 meter-long walkway at their self-selected walking pace for all overground conditions. The calculated overground velocity was then utilized to set the treadmill speed for both treadmill conditions. Participants practiced as needed to allow them to adapt to walking with the weight of the robot for both overground and treadmill conditions. Each participant wore a gait belt and was provided with standby assistance with one-minute seated rests between trials to minimize fatigue. All testing was conducted without subjects wearing any ankle foot orthosis.

### Data analysis

Kinematic and footswitch data were collected at 500 Hz and filtered using a recursive low pass Butterworth filter with cutoff frequency of 6 Hz. Files were exported for further processing in Matlab software (MathWorks, Inc., MA) to analyze hip, knee and ankle kinematic data for each gait cycle in which initial contacts were determined via footswitches. An average of 3 gait cycles were collected per trial for OG and OGR conditions, and 7 gait cycles were collected per trial for TM and TMR conditions. On average, 18 gait cycles were collected for overground conditions and 42 gait cycles for treadmill conditions for each subject. All kinematic data was normalized to percent gait cycle. The average and standard deviations of the normalized gait cycles per condition were used in the statistical analysis. Additionally, footswitch data was utilized to determine percent stance and step time for both paretic and nonparetic sides. A symmetry index (SI) was used to quantify paretic and nonparetic percent stance symmetry in gait [29]:(1)

where 0 ≤ *SI *≤ 1 is the symmetry index and *V*_paretic _and *V*_nonparetic _are paretic and nonparetic percent stance durations, respectively. A lower value of the symmetry index indicates higher symmetry and vice versa with regards to stance durations on paretic and nonparetic sides. In other words, a symmetry index value of zero corresponds to perfect symmetry.

### Statistics

Repeated-measures analysis of variance (ANOVA) tests were conducted to test our hypothesis comparing OG vs. OGR and TM vs. TMR conditions using SAS^® ^software (SAS, Cary, NC). The variables that were compared were paretic and nonparetic kinematics, step time and percent stance. Repeated-measures ANOVA tests were also employed to test the symmetry index. To compare across conditions (overground and treadmill) in a secondary analysis, we employed a post- hoc pair wise comparisons using Tukey's test. The significance level was set at *p *= 0.05 for all tests.

## Results

### Demographic characteristics

Patient demographics are summarized in Table 1. Range of motion (ROM) was measured from 0° using a clinical goniometer. All ten participants were chronic-stage stroke survivors who had suffered their first unilateral stroke from 21 to 146 months prior to enrollment (mean time post stroke of 66 months), were between 43 and 75 years of age (mean age of 63 years) had persistent lower extremity hemiparesis (six left and four right paretic).The paretic limb Modified Ashworth Spasticity scores ranged from 0-1+ for knee flexors, 0-2 for knee extensors, 0-3 for dorsiflexors and 0-2 for plantarflexors. The paretic limb Manual Muscle test scores ranged from 1-5 for hip flexion and extension, 2-5 for knee flexion, 1-5 for knee extension, 0-5 for dorsiflexion and plantarflexion. One subject (#10 in Table 1) was able to walk overground and on the treadmill but, due to substantial weakness in her hip flexors, she was unable to walk with the added robot mass and was thus not included in the results.

**Table 1:** Physical and demographic characteristics of stroke participants.

ID	Age (yr)/gender (M/F)	Paretic (L/R)	Assistive device	**BMI [kg/m**^**2**^**]**	TPS [mos.]	Range of Motion (°)			
						Paretic		Nonparetic^b^	
						ADF	APF	ADF	APF
1	60/M	L	None	26.3	21.6	-23	25	-	-
2	75/M	R	SPC	23.3	146.4	3	34	17	43
3	60/F	R	AFO	22.7	88.8	-22	37	-4	45
4	53/F	R	AFO/SPC	20.9	37.2	-3	26	15	57
5	43/F	L	None	33.0	60.0	0	54	3	69
6	72/M	L	QC	26.3	52.8	-15	28	-	-
7	60/F	L	None	30.0	88.8	-15	50	-10	62
8	68/M	L	None	26.7	18.0	-1	43	-1	54
9	64/F	R	None	27.5	56.4	0	35	15	53
10	73/F	L	SPC	23.9	84.0	-22	26	4	44
**Mean**	**63(4M, 6F)**^**a**^	^**a**^**6 L/4 R**	^**a**^**2 AFOs, 4 canes**	**26.1**	**66.0**	**10.0**	**36.0**	**5.0**	**53.0**
**SD**	**10**			**3.6**	**38.4**	**10.6**	**10.3**	**9.9**	**9.2**

^a^Expressed as distribution.

List of abbreviations- *M*: male, *F*: female; *L*: left, *R*: right; *AFO*: ankle foot orthosis; *SPC*: single point cane; *QC*: quad cane; *BMI*: body mass index; *TPS*: time post stroke; *ADF*, *APF*: active ankle dorsiflexion and plantarflexion, respectively with neutral (90°) subtracted.

^b^Nonparetic range of motion for participants 1 and 6 are marked with dashes to indicate missing data because it was not measured in these two individuals.

### Spatiotemporal gait parameters

**Figure 2:**
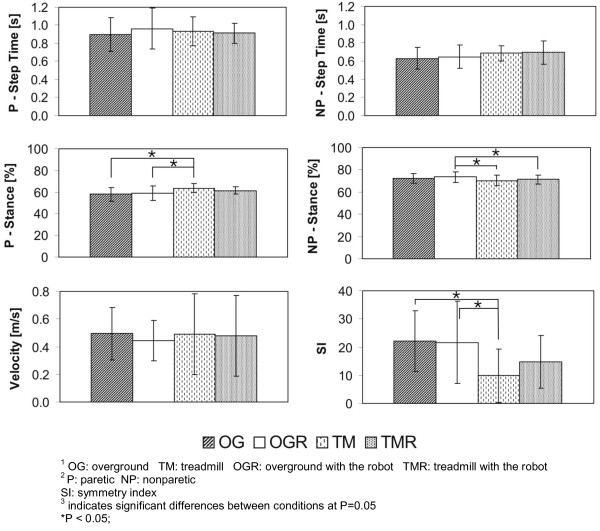
**Graphs for spatiotemporal and symmetry gait parameters**. Average of 9 participants' spatiotemporal and symmetry gait parameters (mean ± SD). These include the step time, stance time, velocity, and symmetry index for the paretic and nonparetic limbs for all four loading conditions (OG no robot, OG with robot, TM no robot, and TM with robot).

Hemiparetic gait parameters for the four conditions are presented in figure 2. The ANOVA tests for our hypothesis resulted in no significant differences between OG and OGR or between TM and TMR conditions except for peak angles (see below). The post-hoc Tukey's test for our secondary analysis which compared across overground and treadmill conditions showed that the percent stance on the paretic side was significantly higher in the TM (63.7 ± 4.4) vs. OG (58.1 ± 6.3 *p *= 0.01) and that the percent stance symmetry was significantly lower for the TM (9.8 ± 9.3) condition compared to the OG (22.1 ± 10.7, *p *= 0.003) condition indicating greater symmetry on the treadmill. The percent stance on the nonparetic limb was significantly higher in the OGR condition (73.5 ± 4.7) compared to the TMR (71.2 ± 3.8 *p *= 0.03) conditions. We also verified that as dictated by our protocol, the measured walking speed did not differ across conditions (OG= 0.496 m/s ± 0.19 m/s, TM = 0.489 m/s ± 0.291 m/s, OGR = 0.442 m/s ± 0.147 m/s, TMR = 0.478 m/s ± 0.292 m/s). There were no other significant differences between conditions.

### Peak angles

**Figure 3:**
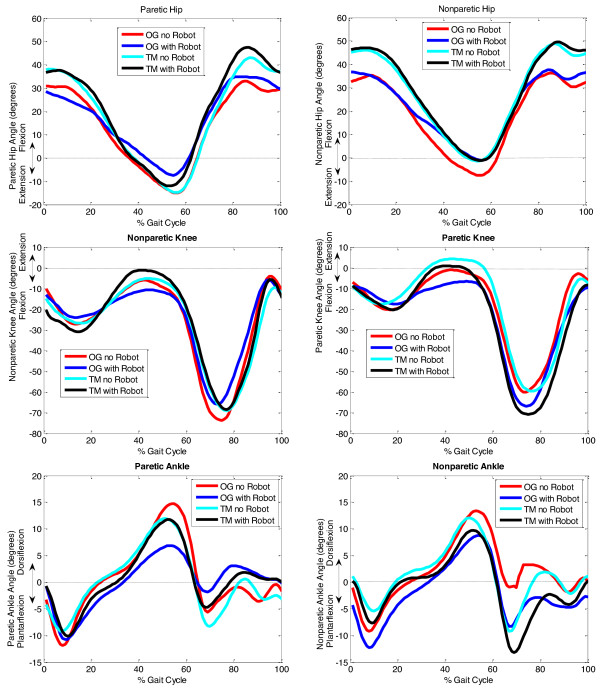
**Gait kinematics**. Gait kinematics (mean ± SD) collected from a single representative subject for the hip, knee, and ankle joints during the four conditions (OG no robot, OG with robot, TM no robot, and TM with robot). For each condition, a total of six (6) gait cycles were averaged. The dashed lines indicate neutral stance taken before the trials.

Figure 3 displays an example of the hip, knee and ankle kinematics among the four conditions for a representative subject. The most notable difference is the significant decrease in maximum paretic dorsiflexion during the OGR condition (3.6° ± 5.5°, *p *= 0.009) compared to the OG (10.4° ± 3.7°, *p *= 0.009) condition. Also, nonparetic maximum knee flexion was greater in the TM (64.1° ± 10.7°, *p *= 0.009) condition compared to the TMR (57.3° ± 12.5°, *p *= 0.009) condition. On the paretic side, maximum hip flexion during the TM condition (35.0° ± 14.0°, *p *= 0.004) was higher compared to the OG condition (25.4° ± 10.9°, *p *= 0.004). Also, paretic maximum hip flexion during the OGR condition (26.0° ± 10.2°, *p *= 0.016) was less than for the TM (35.0° ± 14.0°, *p *= 0.016) and TMR (34.8° ± 13.3°, *p *= 0.016) conditions. Maximum nonparetic hip flexion was greater in the TM (43.9° ± 11.6°, p = .046) condition compared to the OGR (33.7° ± 9.1°, *p *= 0.046) condition. There were no other significant differences among conditions including the relative times at which these peak values occurred in the gait cycle.

## Discussion

These findings support our hypothesis that asymmetric loading of the paretic limb would change bilateral joint kinematics in individuals with hemiparetic gait secondary to stroke. Specifically, the unpowered robot loading reduced nonparetic peak knee flexion on the TM and paretic peak dorsiflexion OG (*p *< 0.05). However surprisingly, mounting the anklebot on the paretic leg leads to no discernable differences in symmetry when walking overground or on the treadmill. It is important to emphasize that the entire study was conducted with the anklebot unpowered. When actively generating training torques at the ankle, these differences due to its added inertia and friction might get even smaller. As for our secondary analysis, similar to others [15], we found that walking on the treadmill showed greater symmetry than walking overground.

Previous work indicated that on average individuals with stroke walked overground at a faster preferred speed than on the treadmill [16]. These results were unlike our findings since we sought to control for differences in speed to allow comparison across conditions. Therefore, the speed in TM and TMR conditions was set to match the OG velocity. The OGR velocity was not directly controlled; however, subjects still walked at a velocity similar to the other conditions.

For the individuals with stroke there were no major dissimilarities in bilateral step times and paretic stance among conditions. The variations in nonparetic stance durations were attributed to the overground and treadmill conditions as opposed to the added loading. The symmetry calculations showed that in the TM condition there was more symmetry between paretic and nonparetic stance phases compared to the OG condition. These findings were similar to previous studies that have shown greater symmetry when walking on a treadmill compared to over ground [3,15,17].

Our kinematic data also showed a 9°-10° increase in paretic maximum hip flexion on the treadmill regardless of loading condition. This difference may be attributable to an increased postural stability obtained due to holding the handrails on the treadmill. Also, there was a significant decrease in paretic maximum hip flexion in the OGR condition compared to both TM and TMR conditions. The ability to produce greater paretic hip flexion in the TMR versus OGR conditions indicates that the treadmill facilitated a greater hip range of motion. This underscores the fact that the added loading was not as influential as the treadmill in affecting these parameters. In contrast, nonparetic maximum knee flexion during the swing phase differed between the two treadmill conditions suggesting that this could have been due to the added robot loading. Furthermore, the robot loading significantly limited paretic ankle dorsiflexion during overground but not in the treadmill training. Our findings are distinct from prior studies where unilateral loading of healthy individuals showed major effect on ankle kinematics while they were walking on a treadmill [30-32]. These differences could be because on the treadmill, stroke volunteers benefited from greater postural support which may have caused the longer paretic side stance durations and altering loading responses observed at the paretic ankle. This could have been due to the repetitive nature of the treadmill task which may have potentially suppressed some of the features characteristic of impaired gait (e.g. circumduction).

Contrary to our findings, previous work has shown that unilateral loading of a limb during treadmill gait does not result in significant differences in hip or knee kinematics [31]. This could be due to a lighter mass (approx. 1.7 kg) used to unilaterally load the limb in [31] which was less than half the mass of our ankle robot (approx. 3.6 kg). In addition, subject demographics could have also contributed to these differences, i.e., participants in [31] included three healthy males whereas all our participants were chronic stroke survivors. Overall, our results appear to suggest that walking on the treadmill with the leg unilaterally loaded or not has little impact on ankle kinematics. Furthermore, these kinematic deviations may be further reduced when the anklebot is used in active mode.

Of interest, not all subjects were able to ambulate with the added mass. Nine of the ten participants were able to ambulate with the added loading and those nine stroke survivors self-reported that they could wear the anklebot comfortably while walking overground and on the treadmill. One of the caveats of the study is that the small sample size is small; therefore, it was difficult to generate an accurate deficit profile for usage (i.e. to determine which individuals with hemiparesis can and cannot tolerate the weight of the ankle robot.)

## Conclusions

In conclusion, the present data suggests that many individuals with hemiparesis can potentially wear an exoskeleton robot safely and with minimum disruption of their unloaded gait pattern.

## List of abbreviations

ANOVA: analysis of variance; AROM: active range of motion; BMI: body mass index; MMSE: mini mental state exam; MMT: Manual Muscle Testing; OG: overground; OGR: overground with the robot; PROM: passive range of motion; SI: symmetry index; TM: treadmill; TMR: treadmill with the robot; UM: University of Maryland; VA: Veterans Affairs.

## Competing interests

Dr. H. I. Krebs holds equity position in Interactive Motion Technologies, Inc., the company that manufactures this type of technology under license to MIT and VA.

## Authors' contributions

IK was involved with data collection and analysis, and drafting of the manuscript. AR, MMR, HIK, RMM, and LWF were involved in contributions to conception and design of the study, interpretation of data, and supervising the manuscript critically for important intellectual content, and AR additionally helped in data collection and analysis. All authors read and approved the final manuscript.
